# Addressing the interaction between food insecurity, depression risk and informal work: findings of a cross-sectional survey among informal women workers with young children in South Africa

**DOI:** 10.1186/s12905-020-01147-7

**Published:** 2021-01-02

**Authors:** Horwood Christiane, Haskins Lyn, Hinton Rachael, Connolly Catherine, Luthuli Silondile, Rollins Nigel

**Affiliations:** 1 Centre for Rural Health, George Campbell Building, Howard College Campus, University of KwaZulu-Natal, Durban, South Africa; 2 RHEdit, Geneva, Switzerland; 3 Department of Maternal, Newborn, Child and Adolescent Health World Health Organization, Geneva, Switzerland

**Keywords:** Working women, Informal economy, Antenatal depression, Postnatal depression, Food insecurity, Maternal health, Child health, South Africa, Africa

## Abstract

**Background:**

There is a high burden of depression globally, including in South Africa. Maternal depression is associated with poverty, unstable income, food insecurity, and lack of partner support, and may lead to poor outcomes for mothers and children. In South Africa one-third of working women are in informal work, which is associated with socioeconomic vulnerability.

**Methods:**

A cross sectional survey explored work setting and conditions, food security and risk of depression among informal working women with young children (0–3 years). Depression risk was assessed using the Edinburgh Postnatal Depression Score (EPDS) and Whooley score. Food insecurity was evaluated using Household Food Insecurity Access Scale. Data was analysed using SPSS and Stata.

**Results:**

Interviews were conducted with 265 informal women workers. Types of work included domestic work, home-based work, informal employees and own account workers, most of whom were informal traders. Most participants (149/265; 56.2%) earned between US$70–200 per month, but some participants (79/265; 29.8%) earned < US$70 per month, and few earned > US$200 per month (37/265; 14.0%). Many participants experienced mild (38/267; 14.3%), moderate (72/265; 27.2%) or severe (43/265; 16%) food insecurity. Severe food insecurity was significantly higher among participants with the lowest income compared to those with the highest income (*p* = 0.027). Women who received financial support from the baby’s father were less likely to be food insecure (*p* = 0.03). Using EPDS scores, 22/265 (8.3%) women were designated as being at risk of depression. This was similar among postnatal women and women with older children. Household food insecurity was significantly associated with depression risk (*p* < 0.001).

**Conclusions:**

Informal women workers were shown to be vulnerable with low incomes and high rates of food insecurity, thus increasing the risk for poor maternal health. However, levels of depression risk were low compared to previous estimates in South Africa, suggesting that informal workers may have high levels of resilience. Interventions to improve social protection, access to health services, and support for safe childcare in the workplace could improve the health and wellbeing of these mothers and support them to care for their children.

## Background

Depression represents a huge burden of disease globally, particularly among women during pregnancy and in the post-partum period [1]. Depression in pregnancy and after childbirth is highly prevalent in low-income countries, especially in high poverty settings [2–4]. In South Africa (SA) estimated rates of depression in the antenatal period range from 22 to 47% [5–7], and several studies suggest that about one-third of postnatal women are at high risk of depression [8–11]. Depression contributes heavily to the overall disease burden in SA [12], and recent evidence suggests that perinatal anxiety disorders are also prevalent [13].

Depression is associated with significant life challenges, including poverty, unstable income, food insecurity, lack of partner support, violence and poor health [9, 10, 14–17]. Maternal depression reduces quality of life and is a leading cause of disability and poor maternal health outcomes, especially in the context of HIV [11, 18]. Further, maternal depression has been associated with adverse effects on the quality of the mother-infant relationship and child development outcomes [4, 19], which can have implications for young children’s long-term psychosocial, cognitive, and economic well-being [10, 11]. Maternal mental health is therefore an important public health concern in SA, however it is frequently neglected and continues to be of low priority in health care practices [3, 7].

Food insecurity is defined as having uncertain or limited availability of nutritionally appropriate food or being unable to procure food in socially acceptable ways [20, 21]. Depression and food insecurity frequently occur together. Food insecurity has been shown to be strongly associated with depression [22], and depression in mothers in low-income households may be a predictor of household food insecurity [23]. Household food insecurity is associated with poor health, development and behavioural outcomes among children [24, 25], and is common in SA, with around 25% of the population considered food insecure [26].

Informal work is frequently associated with vulnerability and poverty, particularly financial and food insecurity [27, 28]. Of the 6.8 million working women in SA, almost 2 million work informally, the largest groups being informal traders, agricultural workers and workers in private homes (domestic workers) [29]. By definition, informal workers do not have access to income protection including unemployment, sickness and maternity benefits. The period during pregnancy and after childbirth is a vulnerable time for all mothers since childcare and household responsibilities often fall disproportionately on women [29]. In SA, female-headed households are common (43%) [30], and it may be especially difficult for mothers in informal work, often already vulnerable due to their working conditions and reduced earning potential, to manage ongoing childcare and household responsibilities [30]. Several studies have shown how poverty and food insecurity interact in a vicious cycle among socially and economically deprived populations, leading to depression and poor health outcomes among women [9, 15].

Thus, factors associated with food insecurity and depression are likely experienced by women working in the informal economy. However, little focus has been given to the multiple risks of depression facing female informal workers and data is sparse among this population group, especially in low- and middle-income settings [16]. Data from one Brazilian study suggests a higher risk of psychological symptoms among women informal workers compared to formal workers living in the same neighbourhood [31].

Women who work in the informal economy in SA as a group are particularly neglected in maternal and child health programmes, and there have been no attempts to address their particular needs [32]. For example, informal workers face barriers that may prevent their seeking antenatal, immunisation or reproductive health care services, but no provision is made for them to attend outside of working hours or be fast-tracked so they can return to work. To address this gap, we conducted this study to explore the interaction between informal work, food insecurity and depression among women with young children working in the informal economy in urban and peri-urban areas of Durban, *KwaZulu-Natal *(KZN) Province, SA.

## Methods

A cross sectional survey methodology was employed to explore the work setting and conditions, income, food security and risk of depression among informal working women with children under the age of 3 years.

### Study setting

The study was conducted in three primary health care (PHC) clinics in three townships in Durban, South Africa. In these areas unemployment is high at around 20%, with high rates of poverty, and an average annual income of R29 400 (USD2000) [33]. In addition, there is poor access to basic services including water and sanitation and informal housing is common, but most households do have access to electricity [34]. A recent study in Durban found that women in the informal economy work long hours and many only earn < R1000 (USD70) per month [27]. Primary caregivers of children living in a low-income household are entitled to an SA government provided Child Support Grant (CSG) of R420 (USD30) per month for each child [35].

Health care for pregnant women and children is provided free of charge in PHC clinics, and there are high rates of antenatal clinic attendance (94%) and facility-based delivery (96%) [36]. HIV prevalence is high among young women in KZN, with 41.1% of pregnant women attending government antenatal clinics testing HIV positive in 2017 [37].

### Recruitment and sampling

Women were recruited while waiting in the queue in the three participating clinics. Trained fieldworkers approached women in the clinic waiting area and explained the study. Eligibility was determined using a structured screening tool based on the definition of informal work set out above (Additional file 1). Eligible participants were informal workers, aged 18 years or older, with a child under the age of 3 years. Informal workers were either informally employed or own account (self-employed) workers. Informally employed women were defined by: (1) receiving money from an employer; (2) having no formal work contract; and (3) not contributing to SA’s mandatory Unemployment Insurance Fund (UIF). Own-account workers provided goods and services directly to customers but were not tax registered or paying tax. Women were excluded from the study if they worked fewer than 3 days per week or had been in informal work for < 6 months in order to focus on those women with longer term experience of informal work.

A stratified sampling approach was employed to recruit equal numbers of eligible women with children aged < 1 year (defined as the postnatal period) and mothers of children aged 12–35 months. Given the well-documented high risk of postnatal depression in SA, this approach was used to determine depression prevalence in the postnatal population compared to the wider population of informally working mothers.

A sample size of 192 women (96 with children aged < 1 year and 96 with children aged 12–35 months) was required to detect a 20% difference in depression risk among postnatal women compared to the general population of informal working mothers. This sample size calculation assumes a 35% rate of depression risk in women with children 1– < 3 years of age with 95% probability and 80% power.

### Data collection

Data collection was undertaken among women waiting in the queue in the three participating clinics. Trained fieldworkers approached women in the clinic waiting area and explained the study. Eligibility was determined using a structured screening tool based on the definition of informal work set out above. Data were collected using a structured questionnaire administered in the local language (IsiZulu) (Additional file 1), and data collection continued until the sample size was reached. All eligible women agreed to participate and the survey was conducted in a separate room in the clinic to ensure privacy.

We recorded the type of work based on the mother’s reported occupation, workplace (home/street/employer’s premises) and working conditions (own account/self-employed/unpaid or paid in kind).

Risk of depression was assessed using two tools validated for use in SA and translated into IsiZulu: the Edinburgh Postnatal Depression Scale (EPDS) [38, 39] and the Whooley score [6]. The EPDS is the most widely used screening tool for depression among postnatal women, and has been widely used and validated in many settings including in South Africa [40], including among populations of non-postnatal women [41] and in high HIV prevalence settings [42]. The EPDS employs a 7 day recall of depressive symptoms including anxiety, tearfulness and poor sleep. A cut-off score of ≥ 13 was used. Although a range of cut-off scores have been used in different settings, this cut-off has been most commonly used in South Africa [3, 6, 9, 10, 39, 43]. The Whooley questions are three questions to assess low mood and lack of interest as well as suicidal ideation. These two tools were used together to allow triangulation of the findings from two screening tools for depression risk.

The USAID Household Food Insecurity Access Scale (HFIAS), adapted and translated to IsiZulu, was used to assess food insecurity. The HFIAS tool is a nine-item experience-based measure with questions about experiences of food insecurity of the past 4 weeks. The tool is designed to be easy to use and applicable to diverse sociocultural settings, and examines food security related to three domains: anxiety about food access; insufficient quality of food; and insufficient quantity of food [44]. The HFIAS can been shown to be broadly applicable across different cultural contexts and has been widely used and validated in Africa, including in South Africa [9, 22, 45]

A professional psychologist, who was IsiZulu-speaking and had experience with both the EPDS and Whooley tools, trained the fieldworkers to assess depression risk using the two tools. The psychologist also supervised data collection to ensure the correct administration of the tools in the field.

### Data analysis

Quantitative analysis was undertaken using SPSS version 24 and Stata V15.1. Background characteristics, feeding and childcare practices, and conditions of informal work are presented as frequencies and proportions.

To describe the types of work undertaken, participants were retrospectively categorised using a series of steps. Firstly, all women who reported they worked from home were categorised as home-based workers. Women were categorised as domestic workers if they reported they worked in a private household undertaking domestic or childcare work. All other women were categorised either as employees if they reported receiving a wage from an employer, or as own account workers if they were self-employed. Sub-categories of own account workers included informal traders and a variety of other own account jobs.

For the EPDS high risk of depression was determined by a score of ≥ 13. Two positive answers were considered a positive score for the Whooley questions. For both tools a positive answer to questions about suicidal ideation was considered high depression risk, regardless of the answers to the other questions.

Household food security was scored on a scale of 0–27, where 27 is the most food insecure, using the established and validated scoring for this tool [46]. For the multivariable analysis the food insecurity index was dichotomised into food secure (none or mild food insecurity) and food insecure (moderate or severe food insecurity), and the EPDS depression score was analysed on a continuous scale and means and standard deviations reported. The analysis was a two-step process. Initially a multi variable logistic model was used to identify factors associated with food insecurity. Due to the over dispersion of the EPDS depression score, a multi-variable negative binomial model including food insecurity was used to identify factors associated with PND score. In both cases only factors significant at *p* < 0.3 in bivariate analysis were included in the multi-variable model. Adjusted and unadjusted odds ratios and 95% confidence limits are reported. Stata V15.1 statistical software was used in the analysis

### Ethical considerations

All participating women provided written informed consent. Screening, consent processes and data collection were conducted in a private area in the PHC clinics. Referral procedures were established with staff at each of the PHC clinics to offer additional clinical and social support to participants as required. All women identified as at risk of depression or with any thoughts of self-harm were immediately referred to clinic staff for further assessment. Follow up support from a study counsellor was offered to all women identified as being  at risk of depression. Women identified as having moderate or severe food insecurity were referred to a clinic-based social worker.

## Results

A total of 265 interviews were conducted with mothers in informal work between December 2018 and March 2019. Just over 50% of participants had completed secondary school and nearly half were in a relationship but not living with their partner. Close to 50% of participants reported themselves HIV positive. Characteristics of participating mothers are shown in Table 1.

**Table 1:** Characteristics of participating mothers

Characteristics of participants	All participants N = 265 (%)
Median age	29 years (IQR 25–34 years)
Population group (Black / African)	265 (100)
Education	
No schooling	2 (0.8)
Primary school Grade 1–7	7 (2.6)
Secondary school Grade 8–11	122 (46.0)
Completed school (Grade 12)	134 (50.6)
Most recent relationship status	
Single	52 (19.6)
Married	22 (8.3)
Separated/divorced/widowed	4 (1.5)
In a relationship, living with partner	61 (23.0)
In a relationship, not living with partner	126 (47.5)
Mother is currently in a relationship with the child’s father	208 (78.5)
Has another child aged < 3 years	19 (7.2)
HIV status	
Mothers reported having had an HIV test	256 (96.6)
Mother reported herself HIV positive	131(49.4)

### Infant feeding and childcare

Due to the stratified sampling design there were similar numbers of participants with children aged < 12 months of age and with children aged between 12–35 months. Most mothers had breastfed their child, however, only 60% of infants < 12 months of age were still breastfeeding. Most mothers (171/265; 64.5%) took time off work following childbirth and were financially supported during this time by the baby’s father (109/265; 41.1%), other family members (72/265; 27.2%), the SA CSG (71/265: 26.8%), and their own savings (21/265; 7.9%).

The duration of leave taken at the time of childbirth was short, with around 60% of mothers returning to work by the time the baby was 3 months old. Over one-third of mothers did not take any time off work (Table 2).

**Table 2:** Duration of breastfeeding, duration of postnatal leave and childcare practices during work hours

Characteristics	< 12 months n = 132 (%)	12–35 months n = 133 (%)	All n = 265 (%)
Baby’s age when mother stopped breastfeeding			
Never breastfed	18 (13.6)	27 (20.3%)	45 (17.0%)
Still breastfeeding	80 (60.6)	15 (11.3)	95 (35.8%)
0 to < 3 months	15 (11.4)	15 (11.3)	30 (11.3)
3 to < 6 months	11 (8.3)	14 (10.5)	25 (9.4)
6 to < 9 months	8 (6.1)	17 (12.8)	25 (9.4)
9 to < 12 months		7 (5.3)	7 (5.3)
12 to < 15 months		17 (12.8)	17 (12.8)
15 to < 18 months		5 (3.8)	5 (3.8)
18 to 24 months		9 (6.8)	9 (6.8)
> 24 months		7 (5.3)	7 (5.3)
Baby’s age when mother returned to work			
Did not take time off work	40 (30.3)	54 (40.6)	94 (35.5)
< 3 months	43 (32.6)	22 (16.5)	65 (24.5)
3 to < 6 months	29 (22.0)	36 (27.1)	65 (24.5)
6 to < 9 months	4 (3.0)	15 (11.3)	19 (7.2)
9 to < 12 months	0	0	0
12 to < 15 months		1 (0.8)	1 (0.4)
15 to < 18 months		1 (0.8)	1 (0.4)
18 to < 24 months		0	0
> 24 months		2 (1.5)	2 (0.8)
Not yet returned to work	6 (4.5%)		6 (2.3)
Missing /mother unsure	10 (7.6)	2 (1.5)	11 (4.5)
Took baby to work initially	26 (19.7)	28 (21.1)	54 (20.4%)
Person who usually cares for child while mother is working			
Child’s grandmother	31 (23.5)	21 (15.8)	52 (19.6%)
Child’s father	2 (1.5)	1 (0.8)	3 (1.1%)
Child’s sibling	1 (0.8)	2 (1.5)	3 (1.1%)
Other relative	28 (21.2)	18 (13.5)	46 (17.4%)
Non-relative	44 (33.3)	69 (51.9)	113 (42.6%)
Mother herself (takes child to work or works from home)	26 (19.7)	18 (13.5)	44 (16.6%)
Place where child is cared for during working hours			
Mother’s residence	76 (57.6)	47 (35.3)	123 (46.4%)
Carer’s home	22 (16.7)	20 (15.0)	42 (15.8%)
Current workplace	3 (2.3)	2 (1.5)	5 (1.9%)
Crèche or school	29 (22.0)	62 (46.6)	91 (34.3%)
Other	2 (1.5)	2 (1.5)	4 (1.5)

### Conditions of informal work

Over 40% of participants had been at the same job between 1 and 3 years, and close to half worked 5–6 days each week. Three-quarters of women reported they worked regular hours, however over 60% did not benefit from a fixed income and were paid depending on the work done. An overview of informal working conditions reported by participants is shown in Table 3.

**Table 3:** Overview of informal work conditions of participants

Conditions of informal work	All workers n = 265 (%)
Length of time in the same job	
Less than 1 year	93 (35.1%)
1–3 years	114 (43.0%)
4 or more years	58 (21.9%)
Number of days worked per week	
3–4 days	94 (35.5%)
5–6 days	119 (44.9%)
7 days	52 (19.6%)
Works in same location every day	208 (78.5%)
Regular hours of work	201 (75.8%)
Work setting	
Mostly indoors	220 (83.0%)
Mostly outdoors with shelter	22 (8.3%)
Mostly outdoors without shelter	23 (8.7%)
Wage is fixed or variable	
Receives a fixed wage	101 (38.1%)
Not fixed / depends on work done	164 (61.9%)
How often receives wages	
Weekly	42 (15.8%)
Monthly	176 (66.4%)
Irregularly	40 (15.1%)
Other	7 (2.6%)

### Types of informal work

Just over 40% of participants reported they worked as employees in informal enterprises, including as cleaners (19), hairdressers (18), waiters (7), shop assistants (24), factory workers (12), office administrators (4), call centre workers (3), and security guards (3). Home based work included hairdressing (23), and making and selling goods, such as crafts, sewing, and baking (9). Other participants were self-employed as informal traders and in a variety of other own account jobs such as crèche work, fashion design, catering, plumbing, welding, sewing and marketing (Table 4).

**Table 4:** Working conditions in different types of informal work

Type of work	Conditions of work (N = 265)					
	Proportion in each category n (%)	Earning < R1000 monthly n (%)	Irregular working hours n (%)	Work 7 days per week n (%)	Paid monthly n (%)	Fixed wage n (%)
Domestic workers	56 (21.1)	12/56 (21.4)	5/56 (8.9)	1/56 (1.8)	49/56 (87.5)	36/56 (64.3)
Home based workers	49 (18.5%)	25/49 (51.0)	29/49 (59.2)	28/49 (57.1)	19/49 (37.8)	1/49 (2.0)
Employed in informal business	116 (43.8)	31/116 (26.7)	20/116 (17.2)	10/116 (8.6)	84/116 (72.4)	60/116 (51.7)
Own account workers: Informal traders	29 (10.9)	10/29 (34.5)	6/29 (20.7)	10/29 (34.5)	16/29 (55.2)	3/29 (10.3)
Own account workers: other	15 (5.7)	1/15 (6.7)	4/15 (26.6)	3/15 (20.0)	8/15 (53.3)	1/15 (6.7)

A comparison of the characteristics of the different categories of informal work are shown in Table 4. Home-based workers were most likely to work 7 days per week and not receive a fixed wage. Employed women and domestic workers benefited from more regular hours compared with other informal workers, however, many still could not rely on a fixed wage.

### Income among informal workers

Most participants (149/265; 56.2%) reported earning between R1 000-3 000 (USD70–USD200) per month for their work. However, many women (79/265; 29.8%) earned < R1 000 (USD70) per month, with the highest proportion being home-based workers. A minority of women earned more than R3000 (> USD200) per month (37/265; 14.0%).

Most participants received additional income from the CSG (208/265; 78.5%), either for one (89; 33.6%), two (74; 27.9%), three (32; 12.1%), or more (13; 7.8%) children. In most cases participants reported that the father of their child was working (194/265; 73.2%) and had provided either money or material items for the baby in the past one month (181/265; 68.7%).

Most participants reported having a bank account (178/265; 67.2%). Despite being low paid, many mothers were saving money monthly (128/265; 48.3%), had a savings plan (123/265; 46.4%) and paid towards a funeral insurance policy (110/265; 41.5%).

### Financial responsibilities

Participants were asked about current financial outgoings for themselves, their child and the household. Most expenses for which the mother was responsible were shared with others (Table 5). Expenses related to the child were frequently shared with the child’s father. Over half of participants reported they did not contribute to household bills or that these costs were shared with other family members. However, most participants reported being responsible for their personal expenses such their own clothing (122/265; 46.0%), toiletries and/or cosmetics (73/265; 27.5%) and paying for their cell phone or airtime (21/265; 7.9%). Some participants reported they regularly financially supported other family members (25/265; 9.4%) including their parents, siblings or other children not living with them.

**Table 5:** Financial responsibilities of participants

Participants’ financial responsibilities	All n = 265 (%)
Household expenses	
Responsible for paying water, electricity, rates, monthly costs	
Mother alone	62 (23.4%)
Shares the cost	62 (23.4%)
Do not pay/not applicable	141 (53.2%)
Who cost is shared with (n = 62)	
Father of the child	27
Other family members	35
Responsible for paying for rent	
Mother alone	50 (18.9%)
Shares the cost	19 (7.2%)
Do not pay/not applicable	196 (74.0%)
Who cost is shared with (n = 19)	
Father of the child	12
Other family members	7
Non-family member	0
Responsible for paying for groceries	
Mother alone	72 (27.2%)
Shares the cost	157 (59.2%)
Do not pay/not applicable	36 (13.6%)
Who cost is shared with (n = 157)	
Father of the child	61
Other family members	95
Non-family member	1
Responsible for transport costs	
Mother alone	207 (78.1%)
Shares the cost	37 (14.0%)
Do not pay/not applicable	21 (7.9%)
Who cost is shared with (n = 37)	
Father of the child	33
Other family members	1
Non-family member	3
Expenses for the child	
Responsible for paying for children’s clothes	
Mother alone	66 (24.9%)
Shares the cost	181 (68.3%)
Do not pay / not applicable	18 (6.8%)
Who cost is shared with (n = 181)	
Father of the child	168
Other family members	10
Non-family member	2
Responsible for paying for child’s food or milk	
Mother alone	56 (21.1%)
Shares the cost	154 (58.1%)
Do not pay / not applicable	55 (20.8%)
Who cost is shared with (n = 154)	
Father of the child	140
Other family members	13
Non-family member	1
Responsible for paying school fees	
Mother alone	72 (27.2%)
Shares the cost	32 (12.1%)
Do not pay / not applicable	161 (60.8%)
Who cost is shared with (n = 32)	
Father of the child	27
Other family members	5
Non-family member	0
Responsible for paying childcare	
Mother alone	89 (33.6%)
Shares the cost	41 (15.5%)
Do not pay/not applicable	135 (50.9%)
Who cost is shared with (n = 41)	
Father of the child	40
Other family members	1
Non-family member	0

### Household food insecurity

A household is considered to be food secure if household members can access sufficient, safe and nutritious foods of their choice without anxiety. Most women had experienced some form of household food insecurity in the last month, and less than half were designated as food secure (112/265; 42.3%). Some participants experienced mild food insecurity (38/267; 14.3%) where they could maintain the quantity of the household food, but the food was less diverse and not their preferred choice. Nearly 30% of participants had moderate food insecurity (72/265; 27.2%) where not only was the food was of lower quality, but *sometimes* the quantity of food had to be reduced by limiting either the number or the size of meals. Further, 16% (43/265) of participants had severe food insecurity where they *often* had to reduce the number or size of meals, or had run out of food, gone to bed hungry, or spent an entire day and night without food.

Severe household food insecurity decreased with increasing income: 19/79 (24.0%) participants with an income < R1 000 were severely food insecure, compared to 22/149 (14.8%) of participants with an income of R1 000–3 000 and 2/37 (5.4%) participants with an income over R3 000. There was a significant higher prevalence of severe food insecurity among the lowest paid compared to the highest (*p* = 0.027).

In multivariable analysis, moderate or severe food insecurity was higher among mothers with an income less than R1 000 per month, and among mothers reporting themselves HIV positive. Mothers who reported receiving financial support from the child’s father were less likely to be food insecure.

### Depression risk

Among all participants, 8.3% (22/265) were identified at risk of depression using the EPDS score cut off ≥ 13. To provide estimates of depression risk in the postnatal period we considered depression risk among mothers with children < 12 months of age (132/265). Of these mothers, 6.8% (9/132) were identified as being at risk of postnatal depression. Among 133 mothers with a child aged between 12 and 35 months, 13 mothers scored high on the EPDS (13/133; 9.8%). There was no significant difference between depression risk among mothers in the postnatal period compared to mothers with older children (*p* = 0.38). Results were similar using the alternative Whooley Score, where a slightly higher percentage of mothers (40/265; 15.1%) were identified as at risk of depression. Again, prevalence of depression risk was similar among mothers with children under 12 months (21/132; 15.9%) and mothers with older children (19/133; 14.3%).

A small number of participants expressed thoughts of self-harm. Using EPDS, 12 mothers (4.5%) reported they had thought of harming themselves in the past 7 days, as compared to Whooley where 10 mothers (3.8%) reported they had thoughts about self-harm in the past 2 weeks.

### Factors associated with high depression risk

In order to determine the relationship between food insecurity and depression, we dichotomised the food insecurity variable as no or mild food insecurity versus moderate or severe food insecurity.

Using the EPDS score as a continuous variable, Table 6 shows after adjusting for possible risk factors there is a significantly higher risk of postnatal depression among mothers living in households where there is moderate or severe food insecurity. However, receiving financial support from the child’s father and working regular hours were associated with a lower average EPDS score. In our study there was no association between risk of depression and HIV status.

**Table 6:** Factors associated with depression risk with EPDS score as continuous variable

N = 265	Mean post-natal depression score			Bivariable			Multivariable		
	n	Mean	SD	OR	95% CI	*p*	OR	95% CI	*p*
Mother's age									
< 30 years	138	5.34	4.94	Ref					
30–47	126	5.73	4.86	1.07	(0.8–1.4)	0.58	na		na
Mother’s education									
< Grade 12	131	6.16	5.39	REF					
≥ Grade 12	134	4.88	4.28	0.79	(0.6–1.0)	0.07	0.85	(0.7–1.1)	0.18
Mother in a relationship with child’s father									
No	57	6.65	4.96	Ref					
Yes	208	5.20	4.84	0.78	(0.6–1.1)	0.11	0.93	(0.7–1.3)	0.67
Father gives money to support the child									
No	84	7.30	5.09	Ref					
Yes	181	4.69	4.58	0.64	(0.5–0.83)	< 0.001	**0.73**	**(0.6–0.9)**	**0.03**
Mother receives child support grant									
No	57	5.84	5.01	Ref					
Yes	208	5.42	4.87	0.93	(0.7–1.30)	0.63	na		
Monthly earning									
< R1000	79	6.27	5.26	Ref					
R2000–2999	149	5.34	4.75	0.85	(0.6–1.1)	0.26	0.90	(0.8–1.3)	0.93
≥ R3000	37	4.59	4.53	0.73	(0.5–1.1)	0.13	1.03	(0.7–1.5)	0.88
Salary									
Not fixed	164	5.52	5.03	Ref					
Fixed	101	5.50	4.69	0.90	0.8–1.3)	0.98	na		
Working hours									
Irregular	64	6.39	5.41	Ref					
Regular	201	5.23	4.70	0.82	(0.6–1.1)	0.17	**0.75**	**(0.6–0.9)**	**0.03**
Stable work									
< 4 years	207	5.57	4.72	Ref					
≥ 4 years	58	5.29	5.52	0.95	(0.7–1.3)	0.74	na		
Self-reported HIV positive									
Negative	117	4.91	4.59	Ref					
Positive	131	6.19	5.19	1.26	(0.9–1.6)	0.08	0.97	(0.8–1.2)	0.80
No answer/no test	17	4.41	3.99	na					
Household Food Insecurity Access Scale (HFIAS)									
Secure/mild insecure	150	3.67	3.93	Ref					
Moderate/severe insecure	115	7.91	5.00	2.15	(1.7–2.8)	< 0.001	**2.09**	**(1.6–2.6)**	**< 0.001**

Significant findings in bold type. Only significant factors in bivariate analysis were included in the multivariate model

## Discussion

Despite the large number of women working informally in SA and throughout the Global South, very little is known about the relationship between informal work, food security and depression among mothers of young children working in the informal economy. This study sought to address this gap to inform the development of strategies to improve maternal and child health and enable more stable livelihoods for these women.

Our findings show that women in informal work were living in poverty, frequently on very low, unstable incomes despite being responsible for many household and personal expenses. Almost one third of women were earning < R1 000 per month, well below the upper bound poverty line for SA, meaning that this amount is insufficient to supply an individual’s basic food and non-food requirements [47]. Types of work, employment conditions and places of work differed among these women, with employed women and domestic workers benefitting from a relatively stable income and work environment compared to self-employed workers, particularly informal market traders. Informal women workers reported high levels of moderate or severe household food insecurity, when compared with the SA average. Most women took very little or no time off work after childbirth, most likely for financial reasons, and this was likely to affect their ability to breastfeed and care for their children. Studies have shown that inability to adequately provide for the households’ food needs is a key indicator of poverty, and is a significant source of anxiety and determinant of poor mental health [22, 48]. More multi-disciplinary interventions are needed to address these intersecting life challenges of informal working women using a cross-cutting approach [49].

The post-partum period is a vulnerable time for many women, one reason for this is the loss of income at the same time as they have the additional expense of providing for a new baby. Although some women who received the CSG experienced food insecurity, many women reported that the grant money they received for their older children helped support them during their maternity leave. This is supported by other studies, which have further shown that receipt of the CSG can reduce hunger, and improve child nutrition [50]. However, although the SA CSG is the largest cash transfer programme in Africa, reaching over 11 million children, the amount is insufficient to ensure adequate dietary diversity for children or protect low-income families from financial shocks [51]. The CSG is a cornerstone of poverty alleviation in SA but access to the grant remains a challenge, particularly for mothers in extreme poverty. Over 10% of women in our study did not receive any grant, and more needs to be done to address this [52]. In addition, payments start several weeks after the baby is born. Therefore, extension of the CSG to cover the antenatal period would improve coverage and be very likely to improve mothers’ ability to take time off work to breastfeed and care for their new baby.

The depression risk among our population was lower than has been found in several other studies in SA [5, 7, 8], and was similar using two different validated screening tools for depression. This relatively low depression risk was found despite women living in contexts of social and economic adversity and food insecurity, that are known to be associated with high rates of depression [15]. Employment is associated with improved self-esteem and being employed is associated with a reduced risk of anxiety and depression [53]. The support that mothers received from their own family, both financial and practical, could also be a factor in protecting women from depression. Another explanation for low rates of depression risk is that informal women workers may have built significant agency and resilience in response to the considerable adversities they have faced in the workplace. The converse may also be true if high levels of resilience enable women to identify and take up opportunities to generate income from informal work. A person displays resilience when they are able to adjust well to significant risk [54]. Although our study did not measure women’s sense of agency or resilience, it appears that informal workers are resourceful and empowered women who may have learned to actively buffer their health from life stressors. Very few studies are available that explain resilience among women in SA, let alone women who work in the informal economy who face many risks. More research is needed to understand, demonstrate and explain this resilience, as well as the role of social support, to inform more effective mental health interventions, particularly for marginalised women [54].

Most mothers relied extensively on practical and financial support from their family, including after childbirth and before their return to work. In particular, we found that receiving financial support from a current partner was an important mitigating factor for both food insecurity and depression. This suggests that the intimate partner relationship is central to women’s well-being, supporting findings from other studies [5]. However, despite being in a relationship with the father of their child, most women were living with their own family. This is common in SA because of the custom for men to pay a substantial bride price (known as “Lebola”) before marriage. As a result, while the child’s father contributed to the child’s expenses, this support did not extend to cover household running costs or women’s personal expenses, leaving women to carry the burden of providing a home for themselves and their child. Thus, these women experienced the ‘‘double-burden’’ of income earning work and managing childcare and household responsibilities. While this is common among women in many settings, it is likely that women working in the informal sector, with few labour protections, are particularly affected [30]. In addition, childcare is often considered to be the mother’s decision and her sole responsibility [55]. Encouraging positive gender and social attitudes towards childcare in the community and in the workplace through increased awareness of the importance of early nutrition and nurturing care, could strengthen the role of society, including social and public services, in supporting and protecting childcare. Clinic staff, community health workers, and local organisations could support awareness campaigns in the community to improve awareness of nurturing care.

Lack of service support and policy frameworks for women working in the informal economy means public authorities, employers and decision-makers currently have no responsibilities to engage with these issues. However, where there are informal workers working in formal work settings, for example undertaking casual cleaning or administrative work, labour regulations should be enforced. While some organizations have been active in advocating for women's rights and livelihoods [56], it will be important to further mobilise worker and women’s rights organisations and key decision-makers to acknowledge or advocate for the rights of informal working women and their children. This includes advocacy for maternity leave agreements, by-laws which support working pregnant women and mothers, the allocation of safer spaces for childcare facilities and the safe-guarding of trading spaces when pregnant women and mothers are away due to childbirth, breastfeeding or caring for their children. Providing social protection to informal working women during the perinatal period, such as through a pregnancy grant, extended CSG or community saving schemes, could safeguard women’s employment during late pregnancy or the early postnatal period, thereby stabilizing incomes and supporting women’s and children’s health, in particular by delaying the time to return to work after childbirth.

The health system also has an important role to play in addressing the interaction between food insecurity, depression, poverty and informal work. First, health services, including mental health care, should be free, available and accessible to informal working mothers and their children. This would require reorienting the health system so PHC opening hours and available services are organised to meet the needs of women working in the informal economy. For example, women start their trading or domestic work well before health facilities commonly open. As a result, women lose income if they attend a clinic, especially if it is a distance away. To enable more responsive, people-centred care, a solution could be to extend the role of community health workers (CHWs) from visiting homes to include visiting public spaces, which are also women’s workspaces.

Second, in addition to clinical services, women need the capacity, including personal and social efficacy, to sustain their livelihoods while ensuring infants are appropriately fed and cared for. Health providers have a critical role to play in providing life-skills training and mentoring support for women to enable them to problem-solve, plan, and make decisions about their lives and caring for their child. In addition, to support early nutrition and nurturing care, infant feeding and responsive childcare training would enable mothers and caregivers to make and implement optimal, evidence-based caregiving choices for their infants and young children.

Lastly, service support and health workforce strengthening are critical. For example, health care providers must have the capacity to confidently and competently provide services to women working in the informal sector and their children, and be supported by systems that invest in their professional development and well-being.

### Strengths and limitations of the study

One strength of this study was the use of two different locally validated tools to assess depression risk, which allowed for triangulation of the findings and strengthened the results. Field workers were trained and supervised by a professional psychologist to ensure consistency and appropriate use of the tools.

However, some limitations must be acknowledged. Although mothers’ risk of depression was identified using screening tools, we did not conduct a full depression assessment to confirm the depression diagnosis. In addition, although the tools used to assess depression and food insecurity had been validated in SA and were translated into isiZulu, it is possible that cultural and language differences may have led to different interpretations of the questions. All participants were aware that referral mechanisms were in place, and participants may have believed that over-reporting indicators of poverty and depression would give them access to additional support. However, we provided a link to referral services and support that is available to all South Africans, including emergency food supplementation and referral to the South African Social Security agency (SASSA). Women who had been informal workers for < 6 months were excluded, so that data from this group, who may have become informal workers as a result of having a baby, were not included.

Although we have data about the proportion of fathers supporting the mother at the time of delivery, the survey did not probe for actual details of expenditure. A form of graded categorisation of contribution amount may have allowed for more analysis of the associations of support with the other outcomes of interest. However, we recognised it was a balance between obtaining meaningful data and participants not finding the questions too intrusive.

## Conclusion

Our study provides unique and important insights into the association between food insecurity, depression, poverty and informal work among women in Durban, SA. The health and livelihoods of women working in the informal economy, and the health and well-being of their infants and young children are compromised by a vicious cycle of vulnerability. This presents a major public health concern, particularly as the high rate of food insecurity greatly increases the risk for poor maternal health and child health and development outcomes [5, 22]. Further research is needed to improve our understanding of these interactions, particularly the role of social support and resilience as a protective factor, and to understand how these interactions compare with women in formal work.

## Supplementary Information

**Additional file 1.** Survey questionnaire.

## Data Availability

The data or material used in the study is available from the corresponding author upon reasonable request.

## Abbreviations

CHWs: Community Health Workers
CSG: Child Support Grant
EPDS: Edinburgh Postnatal Depression Score
HFIAS: Household Food Insecurity Access Scale
KZN: KwaZulu-Natal
PHC: Primary Health Care
SA: South Africa
UIF: Unemployment Insurance Fund
