# Correction to: Addressing the interaction between food insecurity, depression risk and informal work: findings of a cross-sectional survey among informal women workers with young children in South Africa

**DOI:** 10.1186/s12905-021-01182-y

**Published:** 2021-01-20

**Authors:** Christiane Horwood, Lyn Haskins, Rachael Hinton, Catherine Connolly, Silondile Luthuli, Nigel Rollins

**Affiliations:** 1 Centre for Rural Health, George Campbell Building, Howard College Campus, University of KwaZulu-Natal, Durban, South Africa; 2 RHEdit, Geneva, Switzerland; 3 Department of Maternal, Newborn, Child and Adolescent Health, World Health Organization, Geneva, Switzerland

## Correction to: BMC Women’s Health (2021) 21:2 10.1186/s12905-020-01147-7

Following publication of the original article [1], we were notified that the authors’ first and last names had been incorrectly reversed.

The original paper has been corrected.
