# Supplementary material for: Addressing the interaction between food insecurity, depression risk and informal work: findings of a cross-sectional survey among informal women workers with young children in South Africa
Source: BMC Womens Health. 2021 Jan 2;21:2. doi: 10.1186/s12905-020-01147-7 (PMC7777389; doi:10.1186/s12905-020-01147-7)
Supplement: Supplementary file 1 — Additional file 1. Survey questionnaire. [file 12905_2020_1147_MOESM1_ESM.pdf]

PND questionnaire

The baseline questionnaire consists of the following sections

1. **Administration and informed consent**
2. **Mothers information**
3. **Fathers information**
4. **Work information**
5. **Expenses**
6. **Childcare**
7. **HIV**
8. **EDINBURGH POSTNATAL DEPRESSION SCALE (EPDS) & Whoolley depression score**

Section 1: Administration and Informed consent									
A 1.1	Was informed consent obtained?<br>**Uchazeliwe, imvume itholakele?**	1 = Yes				0= No, participant refused to participate<br>➔ ***End interview***			
A 1.2	Site of recruitment<br>**Indawo la kuxoxisanwa khona**								
A 1.3	Tracking number<br>**Inombolo yokulandelela**								
A 1.4	Interviewers name / initials<br>**Igama lomphenyi mibuzo/ Inishiyali**								
A 1.5	Date of interview<br>**Usuku lwenxoxo mibuzo**	D	D	M	M	Y	Y	Y	Y
**Baby's Height and Weight**<br>Now I would like to ask you if I may look at your Road to Health Card. I would like to look at how your baby is growing.<br>**Manje ngicela ukubuka i-Road to Health Card yakho. Ngithanda ukubheka ukuthi umntwana wakho ukhula kanjani.**									
A 1.6	Is the Road to Health Card present?<br>**Ingabe likhona i-card le-Road to Health?**	1 = Yes				0 = No<br>➔ ***Skip to 1.9***			
A 1.7	What is the birthweight recorded on the RTHC?<br>**Sithini isisindo sokuzalwa esiqoshwe kwi-RHTC?**	☐☐.☐☐ KG							
A 1.8	What is the birth length recorded on the RTHC?<br>**Buthini ubude bokuzwala obuqoshwe kwi-RHTC?**	☐☐☐ cm							
	Measure and record today's weight								

A 1.9	**Kala uphinde uqophe isisindo sanamuhlanje**	☐☐.☐☐ KG
A 1.10	Measure and record today's length?<br>**Kala uphinde uqophe ubude banamuhlanje**	☐☐☐ cm

Section 2: Mothers information

I would like to start by asking you some questions about yourself.<br>**Ngizoqala ngokukubuza imibuzo emayelana nawe**									
MI 2.1	What is your date of birth?<br>**Ingabe wazalwa nini?**	D	D	M	M	Y	Y	Y	Y
MI 2 .2	Mothers population group (observe)<br>**Ubuhlanga bukamama (bheka)**	1 = African<br>2 = Indian<br>3 = Coloured<br>4 = White<br>5 = Other							
MI 2.3	Mothers most recent relationship status?<br>**Ingabe ukhona umuntu ozwana naye?**	1 = Single<br>2= Married<br>3 = Separated / divorced / widowed<br>4= In a relationship and living with partner<br>5= In a relationship and not living with partner							
MI 2.4	What is the highest grade you passed at school?<br>**Ingabe iliphi ibanga oliphumelele esikholeni?**	1 = Never attended school<br>2 = Primary school: grade 1 to grade 7<br>3 = Secondary school: grade 8 to grade 11<br>4 = Completed schooling: grade 12							
MI 2.5	What is the youngest child's date of birth?<br>**Ingabe wazalwa nini lo omcane?**	D	D	M	M	Y	Y	Y	Y
MI 2.6	What is the age of your youngest child?<br>**Ingabe ithini iminyaka yomntwana wakho ongaphansi kweminyaka emithathu?**	……………………………months<br>……………………………years							
MI 2.7	Do you have any other children aged less than 3 years?<br>**Ingabe unabi abanye abantwana abaneminyaka engaphansi kwemithathu?**	1= Yes	0= No ➔***SKIP to 2.9***						
MI 2.8	How old is this child (other child aged <3years)?<br>**Ingabe ineminyaka emingaki i(izi)ngane yakho (ezingaphansi kweminyaka emithathu)?**	……………………………months<br>……………………………years							
MI 2.9	What is the name of the index child? (the youngest child)<br>**Lithini igama lomntwana? (omncane)**								
MI 2.10	Are you currently pregnant?<br>**Ingabe ukhulelwe manje?**	1= Yes	0= No ➔***SKIP to3.1***						

MI 2.11	How many months pregnant are you now?<br>**Ingabe zingaki izinyanga manje?**	………………………………..months

Section 3: Father's Information

Now I would like to ask you for information about the father of your youngest child<br>**Ngicela ukubuza imibuzo mayelana nobaba womntwana.**				
FI 3.1	CHECK QUESTION: Is the father of the child still alive<br>**Ingabe ubaba womntwana usaphila?**	1 = Yes	0 = No ➔ ***Skip to 4.1***	-1= don't know➔ ***Skip to 4.1***
FI 3.2	How old is (name's) father?<br>**Uneminyaka emingaki ubaba womntwana?**<br><br>***Record 99 if mother does not know***	…………………..Years		
FI 3.3	Are you still in a relationship with the father of (name)?<br>**Ngabe usenabo ubudlelwano nobaba womntwana?**	1 = Yes	0=No ➔ ***Skip to 3.5***	
FI 3.4	Are you staying in the same house as the father of your baby most nights?<br>**Ngabe uhlala naye ubaba womntwana?**<br>***4 nights or more a week***	1 = Yes	0=No	
FI 3.5	Does the father of the child work?<br>**Uyasebenza ubaba womntwana?**	1 = Yes	0 = No	-1 = Do not know
FI 3.6	How often does the father see the baby?<br>**Ingabe ujwayele ukubona kangaki umntwana ubaba womntwana?**	1= Every day (at least 4 days per week)<br>2= Every week (<4 days per week, but at least once per week)<br>3= Every month (<once a week, but at least once per month)<br>4= Less frequently than once per month<br>5= Never seen the child ➔**Skip to 3.11**<br>-1= Do not know		
FI 3.7	Does the father ever look after the baby while you are away from home?<br>**Ngabe ubaba womntwana uyahlala anakekele umntwana uma wena ungekho?**	1 = Yes	0 = No ➔**Skip to 3.9**	
FI 3.8	How often does the father take care of the baby while you are away from home?<br>**Ingabe ubaba ujwayele ukumnakekela kangaki umntwana uma wena ungekho ekhaya?**	1=never<br>2=sometimes<br>3=regularly (every week or every month)		

FI 3.9	Does the father play with the child? (includes talking to the baby, playing with any toys or smiling and interacting with the baby)<br>**Ingabe ubaba uyadlala nonmtwana (kubalwa ukukhuluma nengane, ukudlala nengane ngamatoyizi noma ukumoyizelela nokuxhumana nengane)?**	1= Yes	0= No
FI 3.10	Does the father ever take the baby to the clinic? (either with you or without you)<br>**Uke ubaba womntwana ahambise umntwana emtholampilo (yedwa noma nawe)?**	1= Yes	0= No
FI 3.11	Has the baby's father given you any money or material items to take care of the baby since he/she was born?<br>**Ubaba womntwana uke wakunika imali noma izimpahla zokunakekela umntwana kusukela ezelwe?**	1 = Yes	0= No ***➔Skip to 4.1***
FI 3.12	Has the baby's father given you any money or material items to take care of the baby in the past one month?<br>**Ubaba womntwana uke wakunika imali noma izimpahla zokunakekela umntwana kulenyanga edlule?**	1= Yes	0= No

Section 4: Work Information

The following questions are about the work that you are currently doing and the work environment. (includes work environment where usually works if she is on maternity leave)<br>**Imibuzo elandelayo imayelana nomsebenzi owenzayo kanye nendawo osebenzela kuyona (kubala nendawo osebenza kuyona uma ukwi – maternity leave)**		
WI 4.1	What type of work do you do currently?<br>**Iluphi uhlobo lomsebenzi owenzayo**<br><br>**njengamanje?**<br><br>***(If two jobs select MAIN job)***<br><br>*Market trader is someone who* ***has a structure*** *provided for them to work at e.g. shelter or table, & trader pays rent.*<br>*Street vendor is someone who works on the side of the road or in a train station/taxi rank etc. but sets up the stall themselves with no structure available*<br>*Any person making goods for sale and then selling them is a street/market trader*	1= waste picker ➔**Skip to 4.3**<br>2= street or informal vendor (cooking) ➔**Skip to 4.3**<br>3= Street or informal vendor (non-cooking) ➔**Skip to 4.3**<br>4= Domestic worker➔**Skip to 4.3**<br>5= Market traders➔**Skip to 4.3**<br>6= Carry goods for other people ➔**Skip to 4.3**<br>7= Home based worker (includes making goods for sale) ➔**Skip to 4.3**<br>8= Hairdresser ➔**Skip to 4.3**<br>9= Shop assistant (informal) ➔**Skip to 4.3**<br>10= Call centre worker ➔**Skip to 4.3**<br>11= Other (specify)
WI 4.2	Specify if other selected or make notes:<br>**Cacisa noma ubhale kabanzi uma kukhethwe - okunye**	……………………………..Write answer
WI 4.3	How long have you been doing this work? (main occupation)<br>**Sekuyisikhathi esingakanani wenza lomsebenzi (lo owenza kakhulu)?**	1= Less than one year<br>2= One to three years<br>3= Four years or more
WI 4.4	What workplace do you usually work from?<br>**Uvame ukusebenzela kuphi?**<br><br>**Select one option-** this should be the workplace where the participant works at her main occupation	1= Own home ➔**Skip to 4.6**<br>2= Own premises (you own or rent) ➔**Skip to 4.6**<br>3= Employers premises eg factory, shop (including employers home) ➔**Skip to 4.6**<br>4= Temporary structure e.g. Container ➔**Skip to 4.6**<br>5= Fixed or temporary stall in a Market ➔**Skip to 4.6**<br>6= Street stall or vehicle or cart or goods on the ground ➔**Skip to 4.6**<br>7= Landfill site or dump site ➔**Skip to 4.6**<br>8= No fixed location (is mobile) ➔**Skip to 4.6**<br>9= Construction site ➔**Skip to 4.6**<br>10= Agricultural/farm ➔**Skip to 4.6**<br>11= Other

WI 4.5	Please specify if other?<br>**Ngicela ucacise uma ukhethe okunye?**	………………………………………	
WI 4.6	How many days do you usually work each week?<br>**Zingaki izinsuku ozisebenzayo evikini?**<br><br>*Prompt if uncertain*<br>*Mukhuthaze ngemibuzo uma engenaso isiqiniseko*	1= 3-4 days<br>2= 5-6 days<br>3= 7 days	
WI 4.7	Do you work in the same location every day or a different location every day?<br>**Ingabe usebenzela endaweni eyodwa izinsuku zonke noma uyashintsha shintsha nsuku zonke?**	1= same location	2 = Different location
WI 4.8	Is the place where you do your work mostly outdoors or indoors?<br>**Ingabe indawo ojwayele ukusebenzela kuyona ingaphandle noma ngaphakathi?**<br>*Read options out for the mother*<br>*Mufundele umama izimpendulo*	1 = Mostly inside<br>2= Mostly outside with shelter<br>3 = Mostly outside no shelter	
WI 4.9	Do you work during the day or during the night? (night is any time between 18H00 and 06H00)<br>**Usebenza emini noma ebusuku?**<br>**(**Ubusuku inoma ingasikhathisini Phakathi kuka 18H00 no 06H00)	1= During the day<br>2= During the night<br>3= During the day and night	
WI 4.10	Are your working hours regular or irregular<br>**Ingabe amahora owasebenzayo ajwayelekile noma awajwayelekile?**	1= Regular (same time every day/week)	2 = Irregular (different times every day/week)
WI 4.11	What job type best describes the work you do?<br>**Oluphi uhlobo lomsebenzi oluchaza kangcono umsebenzi owenzayo?**<br><br>**Choose one option that best describes the work you do**<br><br>*Own account worker- no other people involved*<br>*Employer- if other people are paid (money) to work in your business*<br>*Employee if you receive payment for your work from another person*	1= Employee (receives money from employer)<br>2= Own account worker (you buy and sell or provide a service and keep the money from the customer yourself)<br>3= Not paid/paid in kind ➔**Skip to 4.16**	
WI 4.12	How much do you earn every month?<br>**Ingabe uhola malini ngenyanga?**	1= Less than R1000<br>2= 1000-3000<br>3= More than 3000	
WI 4.13	How can you describe your salary/wage<br>**Ungabe umuholo wakho ubakanjani?**	1= Fixed amount<br>2= Not Fixed/ Depends on work done (number of clients/ goods sold etc)	

WI 4.14	How often do you get paid?<br>**Ingabe uholelwa kanjani?**	1= Weekly ➔**Skip to 4.16**<br>2= Monthly ➔**Skip to 4.16**<br>3= Irregularly ➔**Skip to 4.16**<br>4= Other (specify)	
WI 4.15	Please specify if other<br>**Ngicela ucacise uma ukhethe okunye**	…………………………………….	
WI 4.16	Are you receiving child support grant<br>**Ingabe uyayihola imali yesibonelelo sizongane (child support grant)?**	1= Yes	0= No➔***SKIP to 4.18***
WI 4.17	For how many children do you receive child support grant?<br>**Bangaki abantwana obaholelayo imali ye-grant?**	1= 1 child<br>2= 2 children<br>3= 3 children<br>4=More than 3 children	
WI 4.18	Do you receive any other source of income other than work and social grant money?<br>**Ingabe kukhona la othola khona imali ngaphandle kwemali yomsenenzi neye-social grant?**	1= Yes	0= No<br>➔***SKIP to 5.1***
WI 4.19	If yes, please specify?<br>**Uma kunjalo, ngicela ucacisa?**	……………………………………….	

Section 5: Expenses

I am now going to ask you about what routine monthly expenses you have to pay and whether you are able to share the costs with anyone or whether you alone are responsible.<br>**Manje ngizokubuza mayelana nezindleko zakho zenyanga okumele uzikhokhe nokuthi uyakwazi ukuzihlanganyela nomunye umuntu noma uzikhokhela wena.**				
E 5.1	Who is responsible for paying water/electricity/municipality monthly costs?<br>**Ubani okhokhela amanzi/ugesi/umasipala?**<br>***Give options to the mother***	1= Alone<br>➔***SKIP to 5.3***	2= Share the cost	3= Do not pay/ not applicable<br>➔***SKIP to 5.3***
E 5.2	Who do you share the expenses with?<br>**Ingabe uzisherisha nobani lezindleko?**	1=father of child	2= Other family member	3= Non family member
E 5.3	Who is responsible for paying child/ children's clothes?<br>**Ubani okhokhela izimpahla zengane/zezingane zokugqoka?**<br>***Give options to the mother***	1= Alone<br>➔***SKIP to 5.5***	2= Share the cost	3= Do not pay/ not applicable<br>➔***SKIP to 5.5***

E 5.4	Who do you share the expenses with?<br>**Ingabe uzisherisha nobani lezindleko?**	1= Father of child	2= Other family member	3= Non family member
E 5.5	Who is responsible for paying child's food or milk?<br>**Ingabe ukukhokhela kanjani ukudla noma ubisi komntwana?**<br>***Give options to the mother***	1= Alone<br>***➔SKIP to 5.7***	2= Share the cost	3= Do not pay/ not applicable<br>***➔SKIP to 5.7***
E 5.6	Who do you share the expenses with?<br>**Ingabe uzisherisha nobani lezindleko?**	1=father of child	2= Other family member	3= Non family member
E 5.7	Who is responsible for paying/buying groceries?<br>**Ingabe ubani okhokhela/othenga igrosa?**<br>***Give options to the mother***	1= Alone<br>***➔SKIP to 5.9***	2= Share the cost	3= Do not pay/ not applicable<br>***➔SKIP to 5.9***
E 5.8	Who do you share the expenses with?<br>**Ingabe uzisherisha nobani lezindleko?**	1=father of child	2= Other family member	3= Non family member
E 5.9	Who is responsible for paying rent?<br>**Ingabe ubani okhokhela i-rent?**<br>***Give options to the mother***	1= Alone<br>***➔SKIP to 5.11***	2= Share the cost	3= Do not pay/ not applicable<br>***➔SKIP to 5.11***
E 5.10	Who do you share the expenses with?<br>**Ingabe uzisherisha nobani lezindleko?**	1=father of child	2= Other family member	3= Non family member
E 5.11	Who is responsible for paying school fees?<br>**Ingabe ubani okhokhela imali yesikole (school fees)?**<br>***Give options to the mother***	1= Alone<br>***➔SKIP to 5.13***	2= Share the cost	3= Do not pay/ not applicable<br>***➔SKIP to 5.13***
E 5.12	Who do you share the expenses with?<br>**Ingabe uzisherisha nobani lezindleko?**	1=father of child	2= Other family member	3= Non family member
E 5.13	Who is responsible for paying transport?<br>**Ingabe uyayikhokhela kanjani i-transport?**<br>***Give options to the mother***	1= Alone<br>***➔SKIP to 5.15***	2= Share the cost	3= Do not pay/ not applicable<br>***➔SKIP to 5.15***
E 5.14	Who do you share the expenses with?<br>**Ingabe uzisherisha nobani lezindleko?**	1=father of child	2= Other family member	3= Non family member
E 5.15	Who is responsible for paying childcare?<br>**Ingabe ukukhokhela kanjani ukunakekelwa komntwana?**<br>***Give options to the mother***	1= Alone<br>***➔SKIP to 5.17***	2= Share the cost	3= Do not pay/ not applicable<br>***➔SKIP to 5.17***
E 5.16	Who do you share the expenses with?<br>**Ingabe uzisherisha nobani lezindleko?**	1=father of child	2= Other family member	3= Non family member

E 5.17	Are there any other expenses you are responsible for?<br>**Ingabe zikhona ezinye izindleko onazo?**	1= Yes	0= No ➔***SKIP to 5.19***
E 5.18	If yes, please specify<br>**Uma kunjalo ngicela ucacise**	…………………………………………………….	
E 5.19	Do you have bank account?<br>**Ingabe unayo i-bank account?**	1= Yes	0= No
E 5.20	Are you able to save any money on a monthly basis?<br>**Ingabe uyakwazi ukuseva noma imalini ekupheleni kwenyanga?**	1= Yes	0= No
E 5.21	Do you have savings plan?<br>**Ingabe unawo ama-savings?**	1= Yes	0= No
E 5.22	Do you pay for funeral policies?<br>**Ingabe uyawukhokhela umasingcwabisane?**	1= Yes	0= No

Section 6: Child Care

I would like to ask you some questions about (name) and the care that (name) receives while you are working. **Ngicela ukubuza imibuzo mayelana no(gama) kanye nokunakekelwa kuka(gama) uma usemsebenzini**			
CC 6.1	Did you *ever* breastfeed (name)?<br>**Wake wanceliswa ibele u(gama)?**	1= Yes	0= No ➔***SKIP to 6.4***
CC 6.2	Are you still breastfeeding?<br>**Ingabe usamncelisa ibele?**	1= Yes ➔***SKIP to 6.4***	0= No
CC 6.3	How old was (name) when you stopped breastfeeding?<br>**Wayezinyanga ezingaki u(gama) ngesikhathi uyeka ukumncelisa ibele?**	…………………………………..Weeks<br>…………………………………..Months<br>-1= I do not know	
CC 6.4	Did you take time off when (name) was born?<br>**Uwuyekile umsebenzi ngesikhathi u(gama) esezelwe?**	1= Yes	0= No ➔***SKIP to 6.8***
CC 6.5	How did you support yourself while you were not working?<br>**Wawuziphilisa ngani ngesikhathi ungasebenzi?**<br>**Tick all that apply**	1= Child support grant ➔***SKIP to 6.7***<br>2= Father supported ➔***SKIP to 6.7***<br>3= Other family members supported ➔***SKIP to 6.7***<br>4= Savings ➔***SKIP to 6.7***<br>5= other	
CC 6.6	Please specify if other<br>**Ngicela ucacise uma ukhethe okunye**	……………………………………………………….	
CC 6.7	How old was (name) when you returned to work?	……………………………….Months	

	**Waye neminyaka engaki u(gama) ngesikhathi ubuyela emsebenzini?**	…………………………………Years	
		…………………………………-1= Not sure	
CC 6.8	Did you bring (name) to work with you when you returned to work?<br>**Ngenkathi uphindela emsebenzini ingabe ukeweza no(gama) ukuba usebenze naye?**	1= yes	0= no<br>➔***SKIP to 6.10***
CC 6.9	For how long did you bring (name) to work?<br>**Wamuletha isikhathi esingakanani u(gama) emsebenzini wakho?**	1= < 1months	
		2= 1 to <2 months	
		3= 2months to <3months	
		4= 3 to <4 months	
		5= 4 to <5months	
		6= 5 to <6months	
		7= 6 to < 7months	
		8= 7 to < 8months	
		9= 8 to < 9months	
		10= 9 to < 10months	
		11= 10 to < 11months	
		12= 11 to <12 months	
		13= 12months or more	
		14= Baby still with me at work	
CC 6.10	Does (name) live in the same household as you?<br>(sleeps in the same household as mother at least four nights per week)<br>**Ngabe u(gama) uhlala naye? (ulala ekhaya elilodwa nomama okungenani ubusuku obuwu-4)**	1= Yes<br>➔***SKIP to 6.13***	0= No
CC 6.11	Where is (name) living?<br>**Uhlala kuphi u(gama)?**<br>Note that the mother may be living 4 nights a week away from her own home	1= With the mothers family	
		2= With the father or fathers family	
		3= In your own family/marital home (where mother stays with other children and their father)	
		4= With non-relative	
CC 6.12	How often do you usually see (name)?<br>**Ujwayele ukumbona kangaki u(gama)?**	1= At least once every week ➔**SKIP to 6.14**	
		2 = At least once every month ➔**SKIP to 6.14**	
		3= Less than once a month ➔**SKIP to 6.14**	
CC 6.13	Who usually cares for (name) while you are working?<br>**Ubani ovamise ukunakekela u(gama) uma usemsebenzini?**	1= Childs grandmother	
		2= Childs father	
		3=Childs sibling	
		4= Other relative	
		5 = Non relative	
		6 = Yourself (take child to work/work at home)	
CC 6.14	Where is (name) cared for while you are working?	1= At your current residence (where you sleep at least four nights/week) ➔***SKIP to 7.1***	
		2 = At the carers home ➔***SKIP to 7.1***	

	**Unakekelwa kephi u(gama) uma usemsebenzini?**<br><br>If more than one, select the place where child is most often cared for	3 = In your workplace ➔***SKIP to 7.1***<br>4 = At a crèche or school ➔***SKIP to 7.1***<br>5= Other<br>-1 = Do not know ➔***SKIP to 7.1***
CC 6.15	Please specify if other<br>**Ngicela ucacise uma ukhethe okunye**	…………………………………………………….

Section 7: HIV

Now I would like to ask you questions about HIV. Please remember that everything you tell me is confidential and that you do not have to answer any question that you do not choose to answer. Please just tell me that you prefer not to answer and I will move onto the next question<br><br>**Manje ngizokubuza imibuzo ephathelene negciwne lesandulela ngculazi. Khumbula ukuthi konke ongitshela kona kuyimfihlo futh akuphoqile ukuthi uphendule noma iyiphi imibuzo ongafuni ukuyiphendula. Ngicela nje ungitshele ukuthi awufuni ukuwuphendula ngobe sengidlulela kumbuzo olandelaya.**				
HIV 7.1	Have you been tested for HIV<br><br>**Usuke wahlolela igciwane lesandulela ngculazi?**	1 = Yes	0 = No<br><br>➔***Skip to 8.1***	-3 = Chooses not to answer<br><br>➔***Skip to 8.1***
HIV 7.2	What was the result?<br><br>**Yayithini imiphumela?**	1 = Positive	2 = Negative<br><br>➔***Skip to 8.1***	-3 = Chooses not to answer<br><br>➔***Skip to 8.1***
HIV 7.3	Are you currently taking ante-retroviral treatment (ART)?<br><br>**Njengamanje uyayidla imishanguzo?**	1 = Yes	0 = No	-3 = Chooses not to answer

Section 8: Food insecurity

I would now like to ask you about the food you have available to feed yourself and the rest of your family.

Ngithanda ukukubuza mayelana nokudla onakho ukuba uzondle wena kanye nabomndeni wakho.

No	Question	Response Options		CODE
FI 8.1	In the past four weeks, did you worry that your household would not have enough food?<br>**Kulamasonto amane adlule, ingabe ukewakhathazeka ukuthi ikhaya**	1=Yes	0= No<br>➔***Skip to 8.3***	….|___|

	**lakho angeke libe nakho ukudla okwanele?**			
FI 8.2	How often did this happen?<br>**Kwenzeke kangaki lokhu?**	1= Rarely (once or twice in the past four weeks)<br>2= Sometimes (three to ten times in the past four weeks)<br>3= Often (more than ten times in the past four weeks)		….|___|
FI 8.3	In the past four weeks, were you or any household member not able to eat the kinds of foods you preferred because of a lack of resources?<br>**Kulamasonto amane adlule, ingabe wena noma ubani ekhaya akakwazanga ukudla izinhlobo zokudla enikufunayo ngoba azikho izinsiza?**	1=Yes	0= No<br>➔***Skip to 8.5***	….|___|
FI 8.4	How often did this happen?<br>**Kwenzeke kangaki lokhu?**	1= Rarely (once or twice in the past four weeks)<br>2= Sometimes (three to ten times in the past four weeks)<br>3= Often (more than ten times in the past four weeks)		….|___|
FI 8.5	In the past four weeks, did you or any household member have to eat a limited variety of foods due to a lack of resources?<br>**Kulamasonto amane adlule, ingabe wena noma ubani ekhaya lakhaya lakho niye naphoqeleka ukuthi nidle ukudla okufanayo ngenxa yokweswela izinsiza?**	1=Yes	0= No<br>➔***Skip to 8.7***	….|___|
FI 8.6	How often did this happen?<br>**Kwenzeke kangaki lokhu?**	1= Rarely (once or twice in the past four weeks)<br>2= Sometimes (three to ten times in the past four weeks)<br>3= Often (more than ten times in the past four weeks)		….|___|

FI 8.7	In the past four weeks, did you or any household member have to eat some foods that you really did not want to eat because of a lack of resources to obtain other types of food?<br>**Kulamasonto amane adlulile, ingabe wena noma ubani ekhaya niye nadla ukudla eningathandanga ukukudla ngoba bezingekho izinsiza zokuthola olunye uhlobo lokudla?**	1=Ye	0= No<br>➔***Skip to 8.9***	….|___|
FI 8.8	How often did this happen?<br><br>**Kwenzeke kangaki lokhu?**	1= Rarely (once or twice in the past four weeks)<br>2= Sometimes (three to ten times in the past four weeks)<br>3= Often (more than ten times in the past four weeks)		….|___|
FI 8.9	In the past four weeks, did you or any household member have to eat a smaller meal than you felt you needed because there was not enough food?<br>**Kulamasonto amane adlulile, ingabe wena noma ubani ekhaya niye nadla ukudla okuncane kunalokhu enikudingayo ngoba kungekho ukudla okwanele?**	1=Yes	0= No<br>➔***Skip to 8.11***	….|___|
FI 8.10	How often did this happen?<br>**Kwenzeke kangaki lokhu?**	1= Rarely (once or twice in the past four weeks)<br>2= Sometimes (three to ten times in the past four weeks)<br>3= Often (more than ten times in the past four weeks)		….|___|
FI 8.11	In the past four weeks, did you or any other household member have to eat fewer meals in a day because there was not enough food?<br>**Kulamasonto amane adlule, ingabe wena noma ubani ekjhaya niye nadla ukudla okuncane osukwini ngoba bekungekho ukudla okwanele?**	1=Yes	0= No<br>➔***Skip to 8.13***	….|___|
	How often did this happen?<br>**Kwenzeke kangaki lokhu?**	1= Rarely (once or twice in the past four weeks)		

FI 8.12		2= Sometimes (three to ten times in the past four weeks)<br>3= Often (more than ten times in the past four weeks)		....|___|
FI 8.13	In the past four weeks, was there ever no food to eat of any kind in your household because of lack of resources to get food?<br>**Kulamasonto amane adlule, bekungekho ukudla nhlobo ukudla ekhaya lakho ngoba zingekho izinsiza zokuthola ukudla?**	1=Yes	0= No<br>➔***Skip to 8.15***	....|___|
FI 8.14	How often did this happen?<br>**Kwenzeke kangaki lokhu?**	1= Rarely (once or twice in the past four weeks)<br>2= Sometimes (three to ten times in the past four weeks)<br>3= Often (more than ten times in the past four weeks)		....|___|
FI 8.15	In the past four weeks, did you or any household member go to sleep at night hungry because there was not enough food?<br>**Kulamasonto amane adlule, ingabe wena noma ubani ekhaya waya kolala ebusuku elambile ngoba bekungekho ukudla okwanele?**	1=Yes	0= No<br>➔***Skip to 8.17***	....|___|
FI 8.16	How often did this happen?<br>**Kwenzeke kangaki lokhu?**	1= Rarely (once or twice in the past four weeks)<br>2= Sometimes (three to ten times in the past four weeks)<br>3= Often (more than ten times in the past four weeks)		....|___|
FI 8.17	In the past four weeks, did you or any household member go a whole day and night without eating anything because there was not enough food?<br>**Kulamasonto amane adlule, ingabe wena noma ubani ekhaya wahlala imini Kanye nobusuku bonke engakaze adle noma yini ngoba bekungekho ukudla okwanele?**	1=Yes	0= No<br>➔***Skip to 9.1***	....|___|

FI 8.18	How often did this happen?<br>**Kwenzeke kangaki lokhu?**	1= Rarely (once or twice in the past four weeks)	….|___|
		2= Sometimes (three to ten times in the past four weeks)	
		3= Often (more than ten times in the past four weeks)	

Section 9: EDINBURGH POSTNATAL DEPRESSION SCALE (EPDS)

Please indicate how you have been feeling over the past 7 days by ticking one of the answers below

Ngicela usho ukuthi ubuzizwa kanjani kulezi zinsuku eziwu-7 ezedlule ngokuthi ukhethe impendulo eyodwa ngezansi.

**EPDS 9.1**	In the past 7 days have you been able to laugh and see the funny side of things - how does this compare with your usual self?<br>**Kulezinsuku eziwu-7 ezedlule ubukwazi ukuhleka nokubona amahlaya ezintweni? Kanjani uma uqhathanisa nesimo sakho esijwayelekile?**	1=☐ much as I always could<br>**Yebo, ngokufanayo nezikhathi ezedlule (0)**<br>2=☐ Not quite as much now<br>**Bekungasafani kahle hle nakuqala manje (1)**<br>3=☐ Definitely not so much now<br>**Impela akusafani nakuqala manje (2)**<br>4=☐ Not at all<br>**Akwenzeki lokho nhlobo, nhlobo (3)**
**EPDS 9.2**	In the past 7 days have you been able to look forward to things with enjoyment - how does this compare with your usual self?<br>**Kulezinsuku eziwu-7 ezedlule izinto ubuzibuka ngehlo lokuzithakasela? Kanjani uma uqhathanisa nesimo sakho esijwayelekile?**	1=☐ As much as I ever did<br>**Njengoba kwakwenzeka ekuqaleni (0)**<br>2=☐ Rather less than I used to<br>**Besekwehlile kancane manje kunasekuqaleni (1)**<br>3=☐ Definitely less than I used to<br>**Impela sekwehlile kakhulu manje kunasekuqaleni (2)**<br>4=☐ Hardly at all<br>**Kuqabukela manje ukuthi ngijabulele izinto njengakuqala (3)**
**EPDS 9.3**	In the past 7 days have you blamed myself unnecessarily when things went wrong<br>**Kulezinsuku eziwu-7 ezedlule ngizilahla mina ngecala uma izinto zingahambanga kahle**	1=☐ Yes, most of the time<br>**Yebo, esikhathini esiningi (3)**<br>2=☐ Yes, some of the time<br>**Yebo, ngesinye isikhathi (2)**<br>3=☐ Not very often<br>**Cha, hhayi njalo (1)**

		4=☐ No, never<br>**Cha, bekungakaze kwenzeke (0)**
**EPDS 9.4**	In the past 7 days have you been anxious or worried for no good reason<br>**Kulezinsuku eziwu-7 ezedlule usuke wakhathazeka emoyeni kodwa singekho isizathu salokho**	1=☐ Not, not at all<br>**Cha, bekungakaze kwenzeke (0)**<br>2=☐ Hardly ever<br>**Cha, hhayi njalo (1)**<br>3=☐ Yes, sometimes<br>**Yebo, ngesinye isikhathi (2)**<br>4=☐ Yes, very often<br>**Yebo, esikhathini esiningi (3)**
**EPDS 9.5**	In the past 7 days have you been feeling scared or panicky for no good reason?<br>**Kulezinsuku eziwu-7 ezedlule ubuzizwa uthukile noma wesaba ngaphandle kwesizathu esizwakalayo?**	1=☐ Yes, quite a lot<br>**Yebo, kaningi (3)**<br>2=☐ Yes, sometimes<br>**Yebo, ngesinye isikhathi (2)**<br>3=☐ No, not much<br>**Cha, hhayi kakhulu (1)**<br>4=☐ No, not at all<br>**Cha, akukaze kwenzeke (0**
**EPDS 9.6**	In the past 7 days have you been feeling that things were getting on top of you?<br>**Kulezinsuku eziwu-7 ezedlule ubuzizwa ukuthi izinto ziba ngaphezu kwamandla akho?**	1=☐ Yes, most of the time I haven't been able to cope at all<br>**Yebo, isikhathi esiningi bengingakwazi ukukhona impela (3)**<br>2=☐ Yes, sometimes I haven't been coping as well as usual<br>**Yebo, kwesinye isikhathi bengingakwazi ukukhona njengasemhleni (2)**<br>3=☐ No, most of the time I have coped quite well<br>**Cha, isikhathi esiningi bengikwazi ukukhona kahle nje (1)**<br>4=☐ No, I have been coping as well as ever<br>**Cha, bengikhona kahle njengokwejwayelekile (0)**

**EPDS 9.7**	In the past 7 days have you been so unhappy that you have been having difficulty sleeping?<br>**Kulezinsuku eziwu-7 ezedlule ubungeneme ngalendlela yokuthi ubuze ube nenkinga yokulala?**	1=☐ Yes, most of the time<br>**Yebo, esikhathini esiningi (3)**<br>2=☐ Yes, sometimes<br>**Yebo, ngesinye isikhathi (2)**<br>3=☐ Not very often<br>**Cha, hhayi njalo (1)**<br>4=☐ No, not at all<br>**Cha, bekungakaze kwenzeke (0)**
**EPDS 9.8**	In the past 7 days have you been feeling sad or miserable?<br>**Kulezinsuku eziwu-7 ezedlule ubunokuzizwa uphatheke kabi noma ukhathazekile?**	1=☐ Yes, most of the time<br>**Yebo, esikhathini esiningi (3)**<br>2=☐ Yes, quite often<br>**Yebo, ngesinye isikhathi (2)**<br>3=☐ Not very often<br>**Cha, hhayi njalo (1)**<br>4=☐ No, not at all<br>**Cha, bekungakaze kwenzeke (0)**
**EPDS 9.9**	In the past 7 days have you been so unhappy that you have been crying?<br>**Kulezinsuku eziwu-7 ezedlule ubunokungathokozi ngalendlela yokuthi ubuke ukhale?**	1=☐ Yes, most of the time<br>**Yebo, isikhathi esiningi (3)**<br>2=☐ Yes, quite often<br>**Yebo, bekuvamile (2)**<br>3= ☐ Only occasionally<br>**Izikhashana ezimbalwa (1)**<br>4=☐ No, never<br>**Cha, akukaze kwenzeke (0)**
**EPDS 9.10**	In the past 7 days has the thought of harming yourself occurred to you?<br>**Kulezinsuku eziwu-7 ezedlule uke wafikelwa umcabango wokuzilimaza?**	1=☐ Yes, quite often<br>**Yebo, isikhathi esiningi (3)**<br>2=☐ Sometimes<br>**Ngesinye isikhathi (2)**<br>3=☐ Hardly ever<br>**Kuqabukela (1)**<br>4=☐ Never<br>**Akukaze kwenzeke (0)**

	Explain to the mother that although the question you are going to ask is similar to the previous one but you want to ask a slightly different question.<br>**Chazela umama ukuthi lemibuzo ozoyibuza icishe ifane nalena edlulile kodwa ufuna ukubuza okuhlukile kancane**		
**EPDS 9.11**	In the past 2 weeks have you felt unable to stop worrying or thinking too much?<br>**Kulamasonto amabili adlule ingabe ukewazizwa ungakwazi ukuyeka ukukhathazeka noma ukucabanga kakhulu?**	1= Yes	0= No
**EPDS 9.12**	In the past 2 weeks have you felt down, depressed or hopeless?<br>**Kulamasonto amabili adlule ingabe uzizwe uphansi, udipresekile noma ungenathemba?**	1= Yes	0= No
**EPDS 9.13**	In the past 2 weeks you had thoughts AND plans to harm yourself or commit suicide?<br>**Kulamasonto amabili adlule ingabe uyewafikelwa imicabango noma amaqhinga okuzilimaza noma okuzibulala?**	1= Yes	0= No

Total:

Proceed to SCID if EPDS score $\geq$ 13

Proceed to SCID?

YES YES
